# Genomic Copy Number Variations in the Autism Clinic—Work in Progress

**DOI:** 10.3389/fncel.2019.00057

**Published:** 2019-02-19

**Authors:** Milen Velinov

**Affiliations:** George A. Jervis Clinic, NYS Institute for Basic Research in Developmental Disabilities (IBR), Staten Island, NY, United States

**Keywords:** CNV (copy number variant), autism spectrum disorder, clinical evaluation, genomic medicine, genetic counseling

## Abstract

The development of advanced technology for microarray-based chromosomal studies helped discover increased prevalence of genomic copy number variants (CNVs) in individuals with autism spectrum disorder (ASD). Chromosomal microarray analysis (CMA) is now an important tool for clinical investigations in patients with ASD. While this technology helps identify high proportion of CNV positive individuals among patients with autism, the clinical interpretation of such genomic rearrangements is often challenged by inconsistent genotype-phenotype correlations. Possible explanations of such inconsistencies may involve complex interactions of potentially pathogenic CNV with additional (secondary) CNVs or single nucleotide variants (SNVs). Other involved factors may include gender-specific effects or environmental contributions. Development of risk models for interpreting such complex interactions may be necessary in order to provide better informed genetic counseling to the affected families.

## CNVs in ASD

The completion of the sequence of the human genome in the beginning of this century made it possible to develop comprehensive advanced platforms for genomic studies based on microarray hybridization (Riggs et al., 2014). Chromosomal microarray analysis (CMA) provides an advanced, high resolution chromosomal evaluation, capable to detect submicroscopic chromosomal rearrangements smaller than 100 kb. Using CMA, an increased prevalence of copy number variants (CNVs) were reported in patients with autism. While in the pre-CMA era chromosomal abnormalities were demonstrated in less than 5% of autism spectrum disorder (ASD; Schroer et al., 1998; Lord et al., 2000) with the use of CMA CNVs were reported in up to 11% of ASD patients in early studies (Sebat et al., 2007; Christian et al., 2008; Marshall et al., 2008). These early reports suggested that most apparently pathogenic CNVs in autistic patients occurred *de novo*. The growing consensus that CMA is the most cost-efficient single genetic test for these patients led to the recommendation of CMA as a first-tier testing for ASD in expert guidelines (Manning et al., 2010; Miller et al., 2010; Shen et al., 2010; Schaefer et al., 2013). While CMA is now an accepted initial test for all patients with ASD, there are still controversies regarding the clinical interpreting of many such submicroscopic rearrangements, due to significant inconsistencies in genotype-phenotype correlations.

The initial studies demonstrated higher prevalence of *de novo* CNVs in sporadic autism patients and were thus supportive of their pathogenicity since they were not observed in unaffected family members or in control individuals. However, the studies of multiplex families showed inconsistencies in genotype-phenotype correlations: presence of CNVs in unaffected family relatives of probands or their absence in affected relatives (Christian et al., 2008; Marshall et al., 2008). For instance, in one of these reports out of 41 families with inherited CNVs 21 of the affected siblings were concordant and 12 were not concordant for inheritance of the familial CNV (Christian et al., 2008). These reports also demonstrated high genetic heterogeneity—most CNVs were unique for a specific family. Nevertheless, some recurrent rearrangements were shown to be consistently associated with ASD in unrelated individuals and families. Some of the commonly identified in the early studies recurrent CNVs were duplications 15q11q13, deletions and duplications 16p11.2 and deletions 22q11.2 (Sebat et al., 2007; Christian et al., 2008; Marshall et al., 2008). In addition, genes known to be associated with autism were identified in autism-associated CNVs. Examples of identified affected genes in these initial reports are *OXY* (oxytocin), *SHANK3* and *NLGN4* (Sebat et al., 2007; Christian et al., 2008; Marshall et al., 2008). Currently over 40 recurrent CNVs consistently associated with ASD are identified (Takumi and Tamada, 2018).

## Recurrent CNVs

Some recurrent chromosomal microdeletions associated with ASD, were characterized prior to the CMA advancement using more traditional chromosomal tests. Examples of such rearrangements (microdeletion syndromes) are deletion 15q11-q13 associated with Prader-Willi and Angelman syndromes, deletion 22q11.2 associated with Velo-Cardio-Facial syndrome, deletion 22q13 associated with Phelan-McDermit syndrome. Typically, these previously known microdeletions are larger and are in the ASD category referred to as “syndromic autism” that is, the patients with such abnormalities tend to have congenital anomalies, facial dysmorphisms and other clinical manifestations in addition to autism. These ASD associated microdeletion syndromes almost always include intellectual disability, early developmental delays, muscle hypotonia, or other clinically recognizable manifestations. They are often recognized in the genetic clinic prior to performing molecular studies and may be confirmed with more targeted tests (Harony-Nicolas et al., 2015; Burke and Maramaldi, 2017; Butler et al., 2018; Prasad et al., 2018).

Some of the more recently discovered recurrent CNVs associated with ASD were also found to have specific clinical manifestations in addition to ASD (Bernier et al., 2016; D’Angelo et al., 2016). However, most of them are difficult to suspect on a clinical basis alone due to the variability of the associated clinical features and their occasional presence in unaffected individuals. The recurrent autism associated CNVs do not result in autism in all carriers. Table 1 shows some of the most commonly identified recurrent, autism-associated CNVs, the correspondent prevalence of autism among their carriers and the approximate proportion of unaffected carrier relatives. The table illustrates the reduced penetrance for autism, and generally for cognitive disabilities observed not only in the listed common rearrangements, but also in the majority of any CMA identified submicroscopic rearrangement.

**Table 1:** Common autism associated CNVs.

Chromosomal locus	Genomic coordinates of	Proportion of patients with ASD	Penetrance	Source references
Del 1q21.1	GRCh38/hg38 chr1: 147, 105, 904–147, 922, 392	<10%	Reduced, 67% carrier parents are unaffected	Brunetti-Pierri et al. (2008), Mefford et al. (2008), Haldeman-Englert and Jewett (2011) and Bernier et al. (2016)
Del 15q11.2 (BP1-BP2)	GRCh38: 15: 20,500,000–25,500,000	27%	Reduced, 65% of carrier parents are unaffected	Butler (2017)
Del 15q13.3	GRCh38/hg38 chr15: 30,500,000–32,500,000	11%	Reduced but high, most carrier parents have some neuropsychological manifestations	van Bon et al. (2010) and Lowther et al. (2015)
Del 16p11.2	GRCh37/hg19 chr16: 29,606,852–30,199,855	24%	Reduced but high most carrier parents have some neuropsychological manifestations	Miller et al. (2009); Shinawi et al. (2010) and Hanson et al. (2015)
Del 16p12.2	GRCh37/hg19 chr16: 29,606,852–30,199,855	46%	Reduced but high, most carrier parents have some neuropsychological manifestations	(Girirajan et al. (2010); Girirajan et al. (2015))

## CNV Associated Pathways

Both deletion and duplication of CNVs may result in decreased gene expression by gene disruption. Gene duplications may also lead to gene overexpression. Ultimately, autism associated CNVs lead to abnormal expression of genes associated with neuronal development. *De novo* CNVs that were identified in sporadic autism individuals were found to often include genes associated with dominant or X linked neurodevelopmental disorders (Pinto et al., 2014). These genes converge in networks associated with neuronal signaling, synapse development and chromatin regulations (Pinto et al., 2014; Takumi and Tamada, 2018).

## CNV Interpretation in the Autism Clinic

The purpose of the clinical genetic evaluation of a child with autism is to try answering some of the questions for the patient’s family: Why did this happen? What will happen next? What are the risks for future pregnancies? If a CNV is identified in patient with ASD the following issues are consecutively addressed:

- Is this a recurrent rearrangement that was previously associated with autism?

Identifying such recurrent CNV, previously shown to be associated with autism facilitates the post-test counseling since the abnormality may be safely concluded to be likely associated with the patient’s autism. In most cases when the parents are not affected, such pathogenic abnormalities are *de novo*. Moreover, published studies of patients with such abnormalities may provide further useful information regarding the disease course and possible co-morbidities. Potential problems may occur due to the reduced penetrance of any such recurrent rearrangement (Table 1). Parental testing is indicated in most of these cases in order to determine recurrent risks for future pregnancies. The proportion of individuals affected with autism is taken into account in the genetic counseling of these families.

- If the identified CNV is not recurrent, the most important first question is whether the abnormality is *de novo* or inherited?

The current expert guidelines recommend follow-up chromosomal studies for parents of children with any identified chromosomal rearrangements (Manning et al., 2010; Miller et al., 2010). Parental studies are done in order to help determine if the abnormality is pathogenic and to provide the family with more precise information regarding recurrence risk in future pregnancies. The value of such parental analysis varies with the type of the proband’s CNV. For instance, if the CNV looks likely to be benign (is of small size and does not include genes associated with neurodevelopment) identifying it in an unaffected parent supports its benign nature. On the other hand, if a CNV is likely to be pathogenic (was previously reported in association with autism, includes genes associated with neurodevelopment) its presence in an unaffected parent argues about reduced penetrance and increased risk for future pregnancies.

Parental studies are often not of much help when the abnormality is of truly unknown significance. If such abnormalities are not inherited, the question regarding their pathogenicity remains unanswered. If they are inherited, questions regarding reduced penetrance or mildly affected carrier parent are in place. The clinical geneticist and the genetic counselor often encounter carrier parents with mild behavioral characteristics of autism spectrum or of another mental illness. Therefore, carefully obtained behavioral family history is very important in such circumstances.

- In non-recurrent CNVs, the most important second question is whether the abnormality is pathogenic?

In 2011, the American College of Medical genetics published guidelines for classification of CNVs based on the following criteria (Kearney et al., 2011):

- Overlap with known contiguous gene syndromes
- Size—the larger the size, the more likely for the rearrangement to be pathogenic
- Gene content—inclusion of coding genes and their function
- Database comparison—other reported individuals with similar CNVs in the available databases
- The presence of the observed CNV in the general population

The three main delineated categories of CNVs in the guidelines were “pathogenic,” “uncertain” and “benign.” The uncertain category was subdivided into “likely pathogenic,” “likely benign” and “with uncertain clinical significance.”

A CNV is typically reported as variant of unknown significance (VUS) by the testing laboratory if it is not known to be associated with abnormal phenotype and if it does not include genes know to be associated with genetic conditions. The presence of such abnormality in unaffected parent increases the likelihood for the aberration to be benign. However parental inheritance does not definitely confirm the benign nature of such rearrangement. Further analysis of available databases may help identify other similarly affected individuals with rearrangements in the same chromosomal region. Such useful databases are DECIPHER, ISCA and Autism Chromosome Rearrangement Database (Firth et al., 2009; Riggs et al., 2013,^1^). Finally, review of the function of individual genes included in the region of rearrangement may be also useful. Often after all the above evaluations, conclusion regarding the role of given CNV for the patient’s autism still cannot be made. The role of the geneticist/genetic counselor is to make comprehensive analysis using all available data from parental studies, database analysis and parental phenotype analysis and to make an informed conclusion regarding pathogenicity and recurrence risk, based on all these factors.

- Is there pleiotropic effect?

Many recurrent CNVs associated with autism were also associated with schizophrenia, bipolar disorder and other behavioral disorders (Marshall et al., 2017). The counseling for such abnormalities is especially challenging for the purpose of family planning since the clinical outcomes associated these CNVs may be difficult to predict.

- Is there a need for further genetic studies?

After the mentioned analyses the clinician need to determine if further genetic studies are warranted in a CNV-positive patient. The advance of next generation-based sequencing technologies made whole exome sequencing and more targeted panels for genetic studies widely available. If the rearrangement is a true VUS, next-generation based targeted panel studies or whole exome sequencing should be always considered since such studies may identify a recessive disorder resulting from mutation in gene located in the CNV region that is “unmasked” by the rearrangement (Helbig et al., 2017).

Overall, the interpreting of the clinical significance of a CNV identified in an autistic patient may be challenging and unsatisfactory for the patient’s family. Parental confusion and lack of clear understanding of the test findings classified as VUS had been demonstrated in parental surveys (Kiedrowski et al., 2016). Better understanding of the complex clinical contributions of CNVs and their interaction with other genomic and environmental factors seem to be of importance. Currently the role of the genetics specialist is to discuss to the best of his/her knowledge the implication of the CMA findings and to suggest studies for further clarification of the findings.

## Suggested Mechanisms of the CNVs Variable Phenotypic Effects

The following issues addressed in recent studies may help provide some answers for better clinical interpretation of autism associated CNVs:

A. Additive effect of CNVs, single nucleotide variants (SNVs) or other sequence variants for autism risk

Additive contribution of sequence variants, outside the primary CNV in relevant genes for the overall ASD/cognitive disability risk load was demonstrated in previous reports. Suggested mechanisms for such effects are involvement of genes outside of the CNV or unmasking of recessive disorders affecting genes within the CNV (Helbig et al., 2017; Demily et al., 2018). In a recent study (Pizzo et al., 2018), the authors studied a large cohort of probands and family members with the common 16p11.2 microdeletion. The affected individuals were scored for cognitive impairment and both carrier and non-carrier family relatives were studied for rare sequence variants using whole exome sequencing and Single Nucleotide Polymorphism analysis. Enrichment for rare sequence variants correlated with disease manifestations and severity in the affected individuals. In addition, the load of such “other hits” was higher in families with stronger family history. Finally, the authors showed that such rare secondary sequence variants are likely to affect functions of relevant genes.

The pathogenicity of a given CNV therefore is likely to be modified by aberrations in other genomic regions.

B. Higher mutational burden in females—the “female protective model”

The higher prevalence of male individuals affected with ASD is well known. One possible explanation of such disproportion may be the suggested “female protective effect” for pathogenic genomic variants. An excess burden of deleterious autosomal CNVs in ASD females was previously shown. Moreover, an excess of maternally inherited deleterious CNVs was also demonstrated (Jacquemont et al., 2014; Duyzend et al., 2016). These findings are likely due to gender specific increased tolerance for genomic aberrations in female individuals. Such studies may at least partially explain the higher ASD prevalence in males. For the purpose of clinical evaluations and counseling, this observation suggests that maternally inherited CNVs from unaffected mothers may be more likely to be pathogenic than these inherited from healthy fathers. Another possible associated conclusion may be that a given CNV is more likely to result in ASD symptoms if inherited by a male child. A recent study examined the effects of prenatal exposure to valproic acid in rodent brains (Konopko et al., 2017). Such exposure is previously shown to lead to autistic-like behavior with higher prevalence of manifestations in male animals. The authors showed that female brains have increased expression of certain splice variants of gene *BDNF* in exposed female brains compared to males. These gene splice variants are suggested to confer better neuroprotection. Such gender dependent reaction to harmful agents may also underly the observed female protective effect in humans.

C. CNVs and environmental factors

Environmental factors have been long implicated in the etiology of ASD. Such considered environmental risk factors include maternal or fetal stress during the pregnancy (Gardener et al., 2011; Jones and Van de Water, 2018). A recent study suggests that maternal infection during pregnancy may increase the risk for autism and its severity in CNV positive individuals (Mazina et al., 2015). Environmental factors acting in conjunction with genomic predisposition may be a possible scenario.

## Future Trends

ASD is believed to be the result of impaired gene expression in the fetal brain, affecting genes with role in neurodevelopment, and especially synaptic development and plasticity. Since such aberrant brain development may be the result of multiple interacting factors, more complex analyses may be necessary in order to achieve precise risk estimations, especially regarding phenotype predictions as part of prenatal counseling.

The developing clinical use of whole genome sequencing (WGS) may provide an efficient method to screen for CNVs and SNVs in both coding and non-coding regulatory genomic regions. WGS was already suggested as a first-tier testing for patients with developmental disabilities (Lionel et al., 2018). Advanced technologies for genomic studies may soon provide the opportunity to rapidly assess multiple genomic factors in the same ASD individual. Gene expression profiling currently used as a research tool may soon have application in developmental disability evaluations. While gene expression in brain tissue is too invasive for use in routine evaluations, recent study of cord blood gene expression suggest that such approach may be used to look for aberration in gene expression and its possible correlation with postnatal brain functioning (Breen et al., 2018).

Analysis of environmental factors may also be necessary in order to better predict the risk for ASD. Evaluation of multiple variables may require more complex risk models. Such risk estimating models are already widely used in clinical oncology assays that aim to determine the risk for cancer progression based on multiple molecular markers (Alexander et al., 2012; Gerami et al., 2015). Such models may be also necessary in the complex field of autism genetics.

## Author Contributions

MV wrote the manuscript.

## Conflict of Interest Statement

The author declares that the research was conducted in the absence of any commercial or financial relationships that could be construed as a potential conflict of interest.

## Footnotes

^1^http://projects.tcag.ca/autism/
